# Abnormal expression of paxillin correlates with tumor progression and poor survival in patients with gastric cancer

**DOI:** 10.1186/1479-5876-11-277

**Published:** 2013-11-02

**Authors:** Dong-liang Chen, Zhi-qiang Wang, Chao Ren, Zhao-lei Zeng, De-shen Wang, Hui-yan Luo, Feng Wang, Miao-zhen Qiu, Long Bai, Dong-sheng Zhang, Feng-hua Wang, Yu-hong Li, Rui-hua Xu

**Affiliations:** 1 State Key Laboratory of Oncology in South China, Sun Yat-sen University Cancer Center, Dong Feng East Road, 510060 Guangzhou, P.R. China; 2 Department of Medical Oncology, State Key Laboratory of Oncology in South China, Sun Yat-sen University Cancer Center, Dong Feng East Road, 510060 Guangzhou, P.R. China; 3 Department of Experimental Research, Sun Yat-sen University Cancer Center, Dong Feng East Road, 510060 Guangzhou, P.R. China

**Keywords:** Gastric cancer, Paxillin, Tumor progression, Prognosis

## Abstract

**Background:**

Paxillin (PXN) has been found to be aberrantly regulated in various malignancies and involved in tumor growth and invasion. The clinicopathological and prognostic significance of PXN in gastric cancer is still unclear.

**Methods:**

The expression of PXN was determined in paired gastric cancer tissues and adjacent normal tissues by Western blotting and real-time PCR. Immunohistochemistry was performed to detect the expression of PXN in 239 gastric cancer patients. Statistical analysis was applied to investigate the correlation between PXN expression and clinicopathological characteristics and prognosis in patients. Additionally, the effects of PXN on gastric cancer cell proliferation and migration were also evaluated.

**Results:**

PXN was up-regulated in gastric cancer tissues and cell lines as compared with adjacent normal tissues and normal gastric epithelial cell line GES-1. Overexpression of PXN was correlated with distant metastasis (*P* = 0.001) and advanced tumor stage (*P* = 0.021) in gastric cancer patients. Patients with high PXN expression tended to have poor prognosis compared with patients with low PXN expression (*P* < 0.001). Multivariate analysis demonstrated that PXN expression was an independent prognostic factor (*P* = 0.020). Moreover, ectopic expression of PXN promotes cell proliferation and migration in AGS cells whereas knockdown of PXN inhibits cell proliferation and migration in SGC7901 cells.

**Conclusions:**

PXN plays an important role in tumor progression and may be used as a potential prognostic indicator in gastric cancer.

## Background

Gastric cancer is among the most frequently diagnosed cancer and the second leading cause of cancer-related mortality worldwide [1,2]. In spite of recent improvement in the clinical treatment of gastric cancer, patients with advanced stage of gastric cancer still have poor survival. Surgical resection is still the only curative therapy available for this disease [3]. Under this circumstance, there is an urgent need to better understand the biological mechanism of this neoplasm so as to guide patient management and develop novel therapeutic strategies.

The paxillin (PXN) gene was first identified as a tyrosine-containing protein in cells transformed by the src oncogene [4] and encodes for a focal adhesion molecule of 68 kD [5]. PXN functions as an adaptor protein that coordinates multiple signals from integrins, growth factors and cell surface receptors [6]. By these protein-protein interactions, PXN regulates diverse physiological process, such as gene expression, matrix organization, tissue remolding, cell proliferation and survival, cell motility and metastasis [6-8]. In addition to the interactions with cytoskeleton proteins, PXN could also bind to several oncogenic proteins, such as v-Src, E6 and BCR-ABL [9-13]. Such proteins could use PXN as a docking site or as a substrate to interrupt or mislead the normal adhesion and growth factor signaling pathways that are essential for controlled cellular growth and migration [5]. PXN has been found to be involved in many tumor types. A previous study reported that PXN could inhibit lung cancer proliferation and motility [14]. However, more recent studies found PXN was overexpressed and acted as an pro-oncogene in a variety of tumors, including non-small cell lung cancer, colorectal cancer, prostate cancer and cervical carcinoma [15-20]. In gastric cancer, Li *et al*. reported that PXN (tyr118) phosphorylation was a key factor for fibronectin-stimulated invasiveness of AGS cells [21]. However, the clinicopathological and prognostic role of PXN in gastric cancer is still unclear.

In this study, we detected the PXN mRNA and protein level in 30 paired tumor tissues and adjacent normal tissues and found that PXN was frequently up-regulated in tumor tissues. In addition, the expression of PXN was associated with poor prognosis in a large cohort of 239 patients. Furthermore, ectopic expression/knockdown of PXN could promote/inhibit cell proliferation and migration in gastric cancer cells.

## Materials and methods

### Human tissue specimens and cell lines

This study was approved by the ethics committee of Sun Yat -sen University Cancer Center and written informed consents for using the samples for research purpose were obtained from all the patients before surgery. We collected 239 paraffin-embedded, archived tissue samples from patients who underwent surgery in Sun Yat-sen University Cancer Center (Guangzhou, China) from 2004 to 2008. Moreover, we obtained paired fresh gastric cancer tissues and adjacent nontumorous tissues from 30 of the 239 patients and kept in liquid nitrogen until use. All the patients had a histologically confirmed diagnosis of gastric cancer after resection. Tumor stage was determined according to the 7th edition of the International Union Against Cancer (UICC) on Tumor-Node-Metastasis (TNM) staging system. All the patients were followed-up regularly every three months after surgery with a median follow-up time of 32.5 months (range from 4 to 75 months). All the patients did not receive any pre-operative treatment. The patients who received adjuvant chemotherapy after surgery were based on 5-FU, platinum or taxol regimens. Relevant clinicopathological information including age, gender, tumor size, tumor depth, lymph node invasion, distant metastasis, differentiation status, TNM stage and treatment strategies were obtained from patients’ medical files.

The human gastric cancer cell lines HGC27, SGC7901, BGC823, AGS and MKN28 were obtained from either the RIKEN Cell Bank or the American Type Culture Collection and were cultured with RPMI 1640 medium (GIBCO, Carlsbad, CA, USA) containing 10% fetal bovine serum (FBS, invitrogen, Carlsbad, CA, USA) in a humidified chamber with 5% CO_2_ at 37°C. Human gastric epithelial cell line GES-1 was purchased from the Cell Bank of Chinese Academy of Sciences (Shanghai, China) and was cultured with Dulbecco’s Modified Eagle Medium (GIBCO, Carlsbad, CA, USA) supplemented with 10% FBS, penicillin (100 U/ml), and streptomycin (100 μg/ml).

### RNA extraction and real-time quantitative RT-PCR analysis

Total RNA was extracted from the tissues or cells using Trizol reagent (Invitrogen, Carlsbad, CA, USA) and complementary DNA (cDNA) was synthesized with 2 μg of total RNA by using M-MLV transcriptase (Promega, Madison, WI). Real-time PCR was performed with an ABI PRISM® 7500 Fast Real-time PCR System (Applied Biosystems, CA, USA) and a SYBR Premix Ex Taq™ kit (Takara, Japan); β-actin expression was used as a reference. The following temperature profiles were used: initial heating at 95°C for 10 min, followed by 45 cycles of denaturation at 95°C for 10 s, annealing at 60°C for 10 s, and extension at 65°C for 10 s. The primers used were:

PXN forward: 5′-ACGTCTACAGCTTCCCCAACAA-3′;

PXN reverse: 5′-AGCAGGCGGTCGAGTTCA-3′;

β-actin forward: 5′-TGGATCAGCAAGCAGGAGTA-3′;

β-actin reverse: 5′-TCGGCCACATTGTGAACTTT-3′.Data were analyzed using the 2^-△△ct^ method.

### Western blot analysis

Total cellular proteins were extracted from tissues or cells and separated by SDS-PAGE gels, Western blot analysis was performed according to a standard method as previously described [22]. For immunoblotting of PXN, a rabbit PXN antibody (1:1000, Cell Signaling Technology, USA) was used, and an anti-GAPDH antibody (1:2000; Santa Cruz Biotechnology, USA) was used as loading control.

### Immunohistochemistry (IHC) analysis

IHC analysis of PXN was performed according to a previously described method [23]. Briefly, the formalin-fixed, paraffin-embedded tissue samples were cut into 4 μm slides, dewaxed in xylene, rehydrated with graded of alcohols, and then treated with 3% hydrogen peroxide to block endogenous peroxidase activity. The slides were boiled in 0.01 mol/L sodium citrate buffer (pH 6.0) in a microwave oven to retrieve tissue antigens. Tissue samples were pretreated with 10% normal goat serum to inhibit non-specific staining and incubated at 4°C with a primary antibody (ab#32084, abcam) over night. Tissue sections were then washed with PBST, treated with an anti-rabbit secondary antibody, incubated with streptavidin horseradish peroxidase complex, and finally developed using diaminobenzidine tetrahydrochloride (DAB).

To assess the expression of PXN, we qualified and scored both the extent and intensity of immunoreactivity. In this study, the scores of the extent of staining were evaluated according to the percentage of cells that had positive immunoreactivity in every microscopic field of view: 0, <25%; 1, 25%-50%; 2, 50%-75%; 3, 75%-100%. The scores of IHC intensity ranging from 0 to 3 were determined as follows: 0, negative staining; 1, weak staining; 2, moderate staining; 3, strong staining. By multiplying the scores for extent and intensity, a total score (range, 0 to 9) was achieved. PXN expression level was considered high with scores of ≥ 4 and low with scores of < 4.

### Cell transfection

For overexpression of endogenous PXN, the coding sequence of PXN was amplified and subcloned into the pcDNA3.1 (+) vector (Invitrogen, Carlsbad, CA, USA) according to the manufacturer’ instructions. AGS cells were then transfected with a negative control vector or a PXN expressing plasmid using lipofectamine 2000 (Invitrogen, Carlsbad, CA, USA). To knockdown endogenous PXN expression in cells, small interfering RNA (siRNA) duplex oligonucleotides targeting human PXN mRNA (si-PXN) was obtained from Ribobio (Guangzhou, China). The targeting sequences were: si-PXN#1: GCAGCAACCTTTCTGAACT; si-PXN#2: GTGTGGAGCCTTCTTTGGT. In the present study, we used si-PXN#1 as it could effectively reduced endogenous PXN expression in our preliminary experiments. The target sequence for scrambled siRNA was 5′-GTCTCCACGCGCAGTACATTT-3′. SGC7901 cells were transfected with si-PXN or scramble siRNA according to the manufacturer’s instructions.

### Cell proliferation assays

The 3-(4, 5-dimethylthiazole-2-yl)-2, 5-biphenyl tetrazolium bromide (MTT) assay was performed to test cell proliferation following a method as previously described [24]. Cells were seeded in a 96-well plate at 1 × 10^3^ cells/well, the spectrophotometric absorbance was measured for each sample at 490 nm, all the experiments were performed in triplicate and repeated for 3 times, and the average was calculated.

For the colony formation assay, cells (500/well) were seeded in a six-well plate and cultured for 14 days at 37°C with 5% CO_2_ humidified air. Colonies were stained with 0.1% crystal violet (1 mg/ml) and the numbers of colonies containing more than 50 cells were counted. The experiment was performed in triplicate and repeated for three times.

### *In vitro* cell migration assay

The cell migratory capacity was determined using transwell chambers (BD Biosciences) according to a method previously described [25]. Briefly, cells (1 × 10^5^/well) were suspended in 100 μl serum-free medium and then added to the upper chamber of the inserts, RPMI 1640 medium (GIBCO) containing 10% FBS (500 μl) was added to the lower chamber as the chemotactic factor. After culture for 22 hours, non-migrated cells on the upper surface were removed gently with a cotton swab and cells that migrated to the lower side of the department were fixed and dyed with 0.1% crystal violet. The numbers of migrated cells were calculated by counting five different views under the microscopy. The experiment was performed in triplicate and repeated for three times.

### Statistical analysis

All the data were presented as mean ± SD, a Student t-test or Chi-square test was employed to compare the differences as appropriate. Survival analysis was performed using the Kaplan-Meier method and the log-rank test. Multivariate analysis with Cox proportional hazards model was used to investigate independent prognostic factors. All *P*-values were two-sided, and a *P*-value of < 0.05 was considered statistically significant. Statistical analysis was performed by the SPSS software package (version 16.0, SPSS Inc) or GraphPad prism 5.

## Results

### PXN is up-regulated in gastric cancer tissues and cell lines

**Figure 1:**
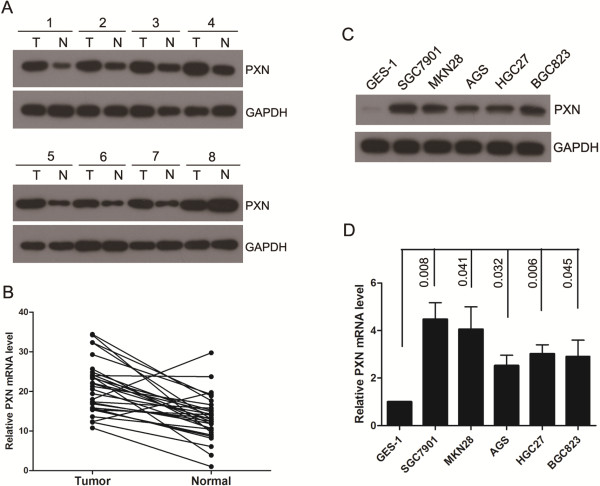
**PXN is up-regulated in gastric cancer tissues and cell lines. A**, PXN protein expression levels in paired gastric cancer tissues and adjacent nontumorous tissues (n = 8). **B**, PXN mRNA expression levels in 30 paired gastric cancer tissues and adjacent nontumorous tissue, (*P* < 0.001). **C**, PXN protein levels in five gastric cancer cell lines and a normal gastric epithelial cell GES-1. **D**, PXN mRNA expression levels in gastric cancer cell lines and a normal gastric mucosa cell line GES-1.

The protein and mRNA level of PXN was evaluated in gastric cancer tissues and cell lines. In paired primary gastric cancer tissues and adjacent nontumorous tissues, Western blot analysis revealed overexpression of PXN in cancer tissues compared with adjacent nontumorous tissues (Figure 1A). The level of PXN mRNA was increased in 27 of 30 (*P* < 0.001) cancer tissues in comparison with that of nontumorous tissues (Figure 1B). Moreover, Western blot analysis showed the expression levels of PXN were higher in gastric cancer cell lines than that of normal gastric epithelial cell line GES-1 (Figure 1C). Consistent with the level of protein, real-time PCR analysis confirmed PXN mRNA level was elevated in all five gastric cancer cell lines (Figure 1D).

### Overexpression of PXN is associated with adverse tumor phenotype and poor prognosis in gastric cancer

**Figure 2:**
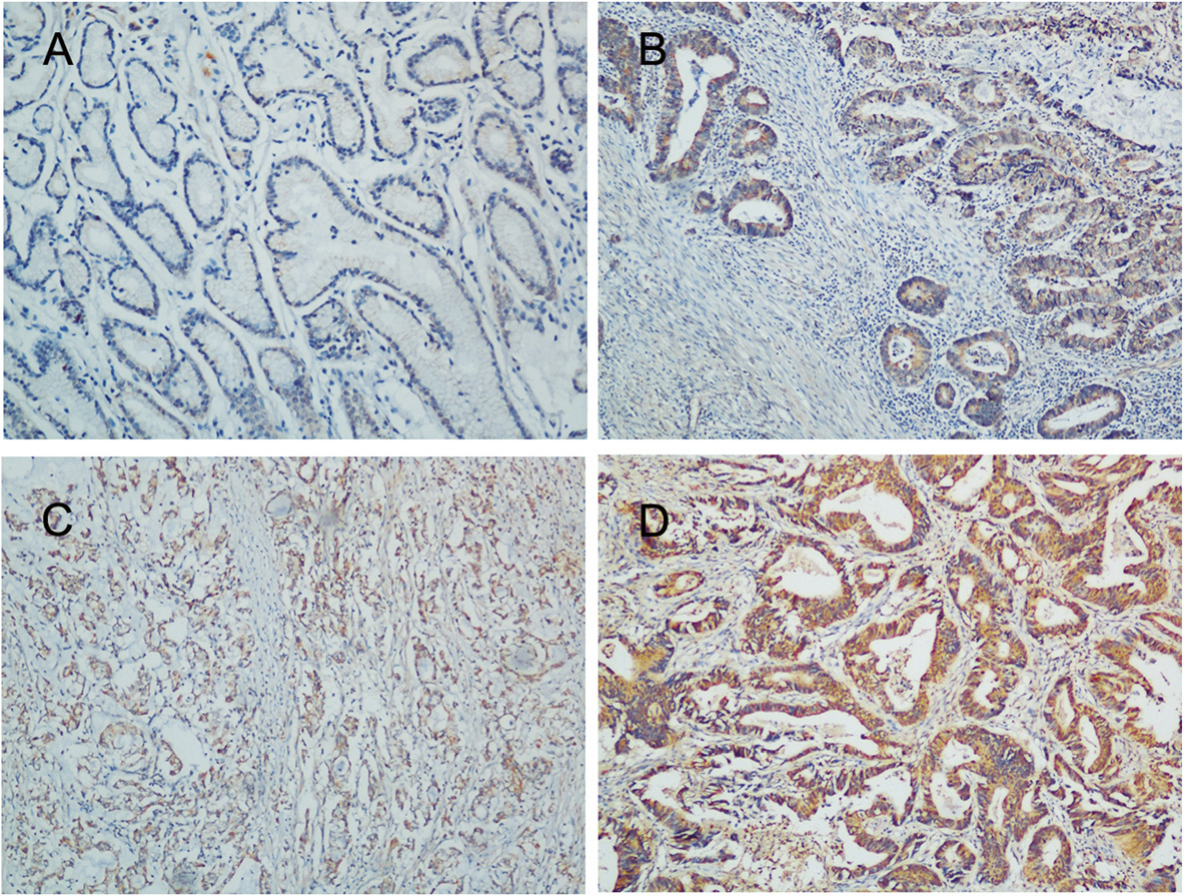
**IHC analyses of PXN expression in gastric cancer tissues, the representative ones are shown. A**, negative staining of PXN in normal gastric mucosa; **B**, weak staining of PXN in well differentiated gastric cancer tissues; **C**, moderate staining of PXN in gastric cancer tissues; **D**, strong staining of PXN in gastric cancer tissues, amplification (×200)

**Figure 3:**
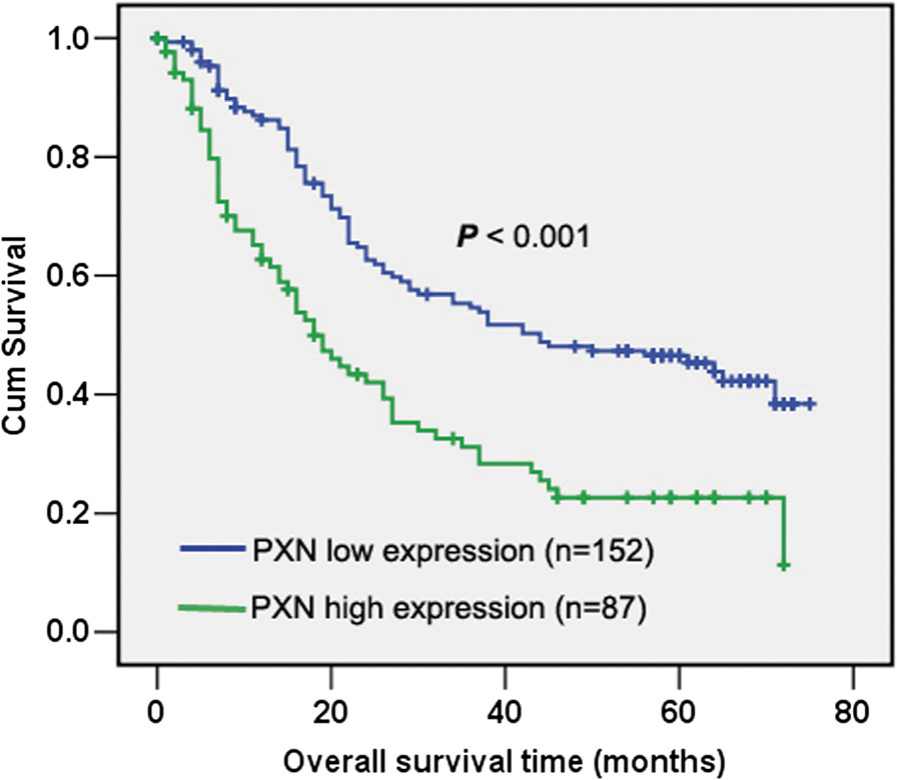
**The prognostic role of PXN in gastric cancer patients.** Kaplan-Meier analysis of overall survival based on PXN expression in all 239 patients, patients with high PXN expression (n = 87) possessed with significantly poor overall survival compared with that of patients with low PXN expression (n = 152) (*P* < 0.001).

We further investigated the expression of PXN in 239 paraffin-embedded gastric cancer tissues by IHC. As shown in Figure 2, PXN protein was mainly located in the cytoplasm of tumor cells. Positive staining was detected in 186 of 239 (77.8%) patients. One hundred and fifty-two patients were possessed with low PXN expression while the other eighty-seven with high PXN expression based on the IHC scores. The associations between PXN expression and clinical and pathologic parameters were summarized in Table 1. High PXN expression was significantly correlated with distant metastasis (*P* = 0.001), TNM stage (*P* = 0.021) and survival status (*P* = 0.004). However, there was no association between PXN expression level and age, gender, tumor depth, differentiation status and treatment. Kaplan-Meier analysis with log-rank test was employed to investigate the prognostic role of PXN for gastric cancer patients. Patients with high PXN expression had significantly worse overall survival than patient with low PXN expression (Figure 3). In addition, Univariate analysis revealed high PXN expression is a risk factor of death in gastric cancer patients (HR = 2.05, 95%CI, 1.47-2.86; *P* < 0.001, Table 2). Besides, other factors including lymph node invasion, distant metastasis and TNM stage were also correlated with overall survival as demonstrated by univariate analysis (Table 2). However, age, gender, differentiation status, treatment and tumor depth were not prognostic indicators in this study. Multivariate analysis indicated only distant metastasis and PXN expression were independent prognostic factors for gastric cancer patients (*P* < 0.001 and *P* = 0.020, respectively, Table 2).

**Table 1:** Correlations between PXN expression and clinicopathological characteristics in gastric cancer patients

		**PXN expression**		
**Characteristics**	**Total No.**	**Low No. cases (%)**	**High No. cases (%)**	***P *** **value**
Age				0.742
< 60	116	75(49.3)	41(47.1)	
≥ 60	123	77(50.7)	46(52.9)	
Gender				0.096
Male	177	118(77.6)	59(67.8)	
Female	62	34(22.4)	28(32.2)	
Tumor depth				0.527
T1	11	9(5.9)	2(2.3)	
T2	29	20(13.2)	9(10.3)	
T3	176	109(71.7)	67(77.0)	
T4	23	14(9.2)	9(10.4)	
Lymph node invasion				0.656
N0	55	35(23.0)	20(22.9)	
N1	85	58(38.2)	27(31.0)	
N2	64	39(25.6)	25(28.7)	
N3	35	20(13.2)	15(17.4)	
Distant metastasis				0.001^a^
M0	173	121(79.6)	52(59.7)	
M1	66	31(20.4)	35(40.3)	
Differentiation status				0.388
Well	99	68(44.7)	31(35.6)	
Moderate	92	55(36.1)	37(42.5)	
Poor and others	48	29(19.2)	19(21.9)	
TNM^b^ stage				0.021^a^
I	28	20(13.1)	8(9.1)	
II	37	23(15.1)	14(16.1)	
III	100	72(47.3)	28(32.2)	
IV	74	37(24.5)	37(42.6)	
Treatment				0.386
Surgery only	116	77(50.7)	39(44.8)	
Surgery + chemotherapy	123	75(49.3)	48(55.2)	
Survival status				0.004^a^
Alive	98	73(48.0)	25(28.7)	
Dead	141	79(52.0)	62(71.3)	

^a^*P* **<** 0.05, Chi-square test; ^b^TNM: T, tumor; N, lymph node; M, distant metastasis.

**Table 2:** Univariate and Multivariate analysis of various potential prognostic factors in gastric cancer patients

**Factors**	**Univariate analysis**		**Multivariate analysis**	
	**HR**^**b**^**(95%CI**^**c**^**)**	***P***	**HR**^**b**^**(95%CI**^**c**^**)**	***P***
Age	1.27(0.91-1.77)	0.154	-	-
Gender	1.11(0.76-1.61)	0.575	-	-
Differentiation	1.42(0.98-2.06)	0.061	-	-
Treatment	1.09(0.71-1.75)	0.456	-	-
Tumor depth	1.32(1.08-1.60)	0.089	-	-
Lymph node invasion	2.05(1.46-2.86)	0.005^a^	1.04(0.75-1.46)	0.803
Distant metastasis	2.74(1.89-3.98)	<0.001^a^	2.51(1.58-3.99)	<0.001^a^
TNM stage	1.64(1.22-2.13)	<0.001^a^	1.32(0.91-1.93)	0.138
PXN expression	2.05(1.47-2.86)	<0.001^a^	1.29(1.04-1.61)	0.020^a^

^a^*P* **<** 0.05.

^b^HR: hazard ratio.

^c^CI: confidence interval.

### PXN promotes tumor growth and proliferation *in vitro*

**Figure 4:**
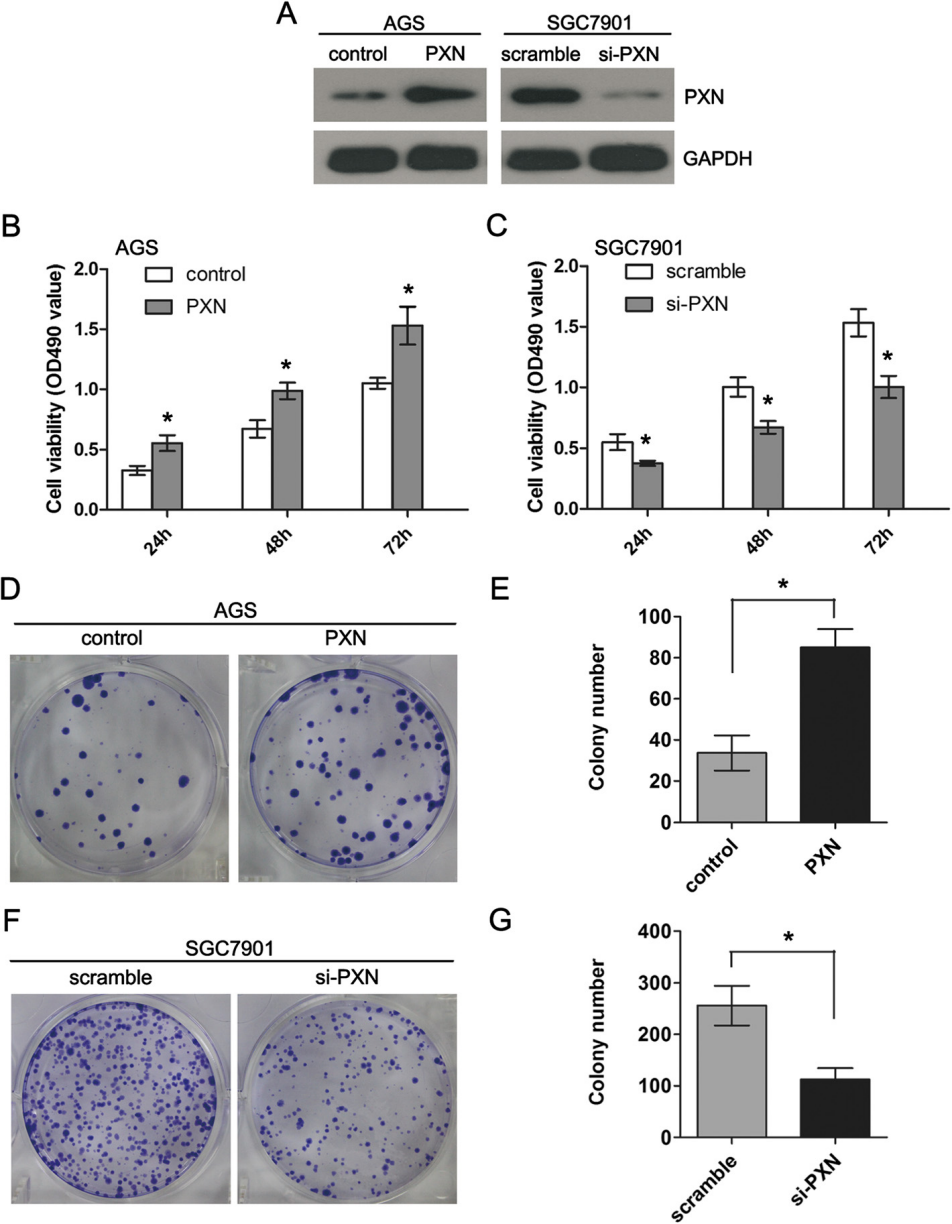
**PXN promotes cell proliferation and colony formation in gastric cancer cells. A**, PXN protein level is elevated after over-expression of PXN in AGS cells and reduced after knockdown of PXN in SGC7901 cells. **B** and **C**, Ectopic expression of PXN promotes cell proliferation in AGS cells (*P* = 0.017) whereas knockdown of PXN inhibits cell proliferation in SGC7901 cells as determined by MTT assays (*P* = 0.019). **D** and **E**, Ectopic expression of PXN stimulates colony formation in AGS cells (*P* = 0.026). **F** and **G**, Knockdown of PXN expression inhibits colony formation in SGC7901 cells (*P* = 0.011).

We further investigated the role of PXN on the tumor cell growth and proliferation through gain-of-function and loss-of-function analysis. AGS cells were transfected with pcDNA3.1(+)-PXN to overexpress PXN (Figure 4A). The effects of PXN on cell growth and proliferation were then evaluated by MTT and colony formation assays. The results showed that ectopic expression of PXN significantly promoted the viability of AGS cells (Figure 4B, *P* = 0.017). The colony formation assay also indicated the number of colonies was markedly increased in PXN overexpressing cells compared with control cells (Figure 4D and E, *P* = 0.026). The impact of PXN on cellular proliferation was further confirmed by performing MTT and colony formation assays after PXN knockdown in SGC7901 cells (Figure 4A). As indicated in Figure 4C, F and G, compared with that of control cells, knockdown of PXN significantly inhibited the growth rate and colony formation ability of SGC7901 cells (*P* = 0.019 and *P* = 0.011, respectively).

### PXN promotes cell migration *in vitro*

**Figure 5:**
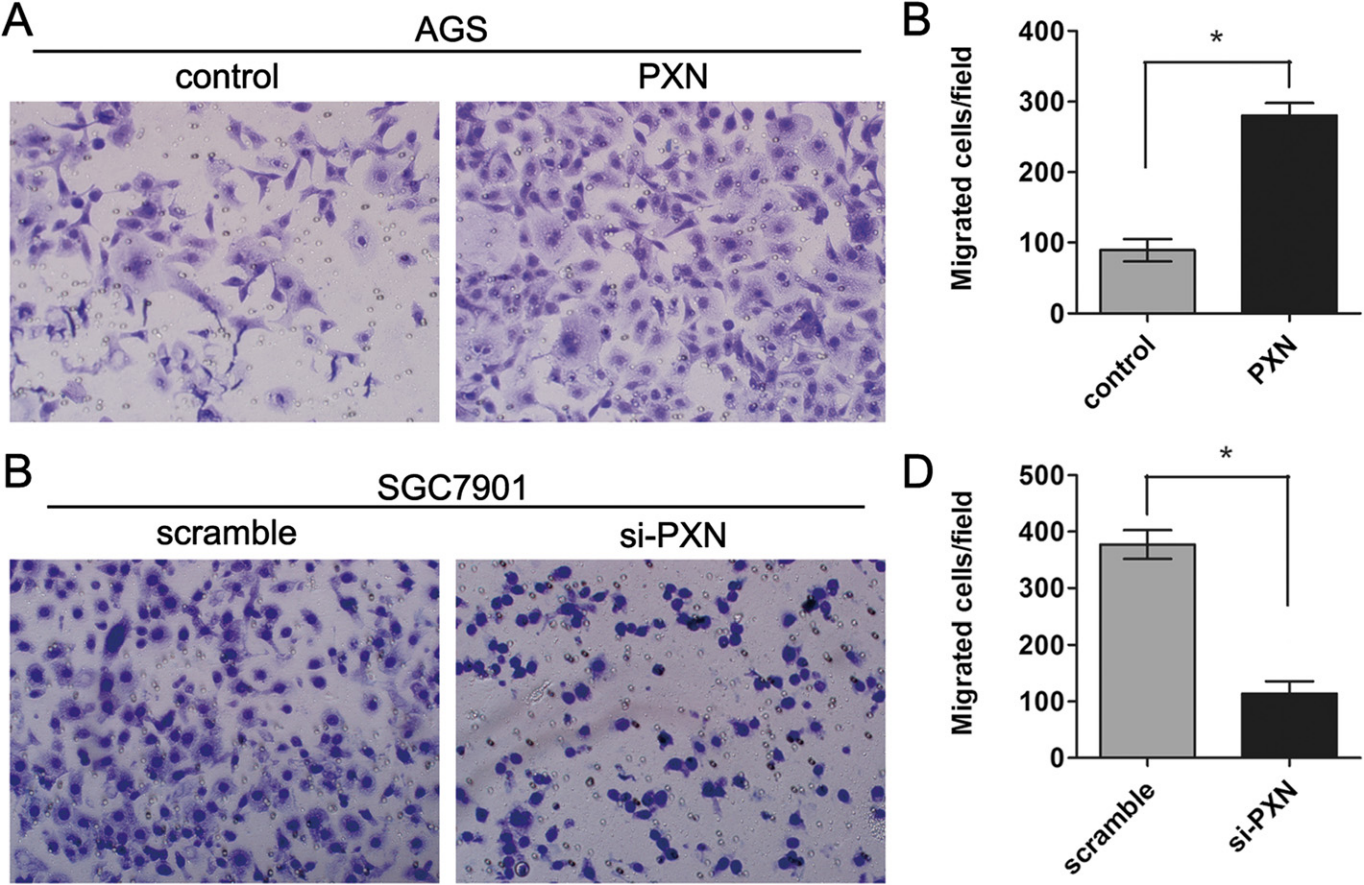
**PXN promotes gastric cancer cell migration *in vitro. *A** and **B**, Ectopic expression of PXN promotes cell migration in AGS cells as demonstrated by transwell assays (*P* = 0.013). **C** and **D**, Knockdown of PXN expression inhibits cell migration in SGC7901 cells (*P* = 0.032), amplification (×100).

As PXN expression is associated with distant metastasis in gastric cancer patients, we then evaluated the potential role of PXN on cellular migration by transwell assays. AGS cells were transfected with PXN overexpressing or control plasmid and seeded in the chamber, and their migratory abilities were determined 24 hours later. The results showed ectopic expression of PXN significantly increased the migratory capacity of AGS cells (Figure 5A and B, *P* = 0.013). On the contrary, knockdown of PXN dramatically reduced the migrated cell number of SGC7901 (Figure 5C and D, *P* = 0.032).

## Discussion

We have previously found that PXN is up-regulated in colorectal cancer and associated with aggressive tumor phenotypes [26]. However, the potential clinical and pathologic role of PXN in gastric cancer is still unknown. In this study, firstly, we detected the expression of PXN in gastric cancer tissues and cell lines. The results showed that PXN was frequently up-regulated in gastric cancer tissues and cell lines compared with adjacent nontumorous tissues or normal gastric mucosa cells. In addition, in a large cohort of gastric cancer patients, overexpression of PXN was significantly associated with aggressive tumor characteristics, such as distant metastasis and advanced TNM stage. Moreover, high expression of PXN was correlated with poor overall survival. Multivariate analysis demonstrated that PXN expression was an independent prognostic factor for gastric cancer patients. These results demonstrated that overexpression of PXN was commonly observed in gastric cancer. PXN might serve as an useful prognostic indicator for patients.

Several studies have reported the clinical and pathological significance of PXN in other tumor types. For example, up-regulation of PXN is found in non-small cell lung cancer [15] and prostate cancer [27]. Expression of PXN is associated with adverse pathologic characteristics in hepatocellular carcinoma [28]; PXN protein level is correlated with advanced clinical stage and distant metastasis in salivary adenoid cystic carcinoma [29]; amplification of PXN is frequently observed in high risk lung cancer [17]. Our findings in gastric cancer are in lines with these results. However, further studies are needed to confirm the clinical role of PXN in gastric cancer.

PXN is a focal adhesion molecule that involved in signaling transduction and cellular migration, it can increase the adhesion between tumor cells and the surrounding extracellular matrix and molecules, thereby promotes or inhibits the tumor cell motilities. The effect of PXN on cell spreading is mainly regulated by tyrosine/serine phosphorylation [30,31]. For instance, in breast cancer, cell migration and invasion stimulated by breast tumor kinase (Brk) is mediated through PXN phosphorylation [32], PXN is the substrate of Brk and functions as a “platform” of Brk. In colon cancer, phosphorylation of PXN at tyrosines 31 and 118 site is essential for pressure-induced cellular metastasis and adhesion [33]. In this study, we found that ectopic expression of PXN stimulated tumor proliferation and migration whereas knockdown of PXN suppressed cellular growth and motility in gastric cancer cells. Our results are similar to what observed in colorectal cancer, in which Jun *et al.* found that ectopic expression of PXN could increase cell migration, invasion and adhesion abilities whereas knockdown of PXN expression by small interfering RNA suppressed these capacities [19]. Taken together, these results suggest that PXN plays an important role in the growth and metastasis of gastric cancer.

## Conclusions

In conclusion, our study revealed a cell adhesion protein PXN is frequently up-regulated in gastric cancer tissues and cell lines. Overexpression of PXN is associated with aggressive tumor phenotypes and adverse overall survival, thus implicating PXN might serve as an useful prognostic indicator. Ectopic expression/knockdown of PXN promotes/inhibits tumor growth and migration, which indicates that PXN may play an important role in the progression and metastasis of gastric cancer. However, further study is needed to investigate the underlying mechanism involved in PXN regulation in gastric cancer.

## Abbreviations

PXN: Paxillin; TNM: Tumor, lymph node, distant metastasis; IHC: Immunohistochemistry; MTT: 3-(4, 5-dimethylthiazole-2-yl)-2, 5-biphenyl tetrazolium bromide; siRNA: Small interfering RNA.

## Competing interests

The authors declare that they have no competing interests.

## Authors’ contributions

CDL conceived of the study, performed the Western blotting analysis, IHC analysis, molecular studies and drafted the manuscript. WZQ, RC, ZZL and WDS collected the clinical data and tissue samples. LHY, WF, QMZ, BL, ZDS, WFH and LYH performed the statistical analysis. XRH participated in the design of the study and helped to draft the manuscript. All authors read and approved the final manuscript.
